# 

**Published:** 1880-07

**Authors:** 


					﻿SURG GENLS OFFS
Vol. XVI.
July, 1880.
No. 2.
0M
ISTOURY.
the u syudk lfdywh MD
E ,Q I T Q R
JLÌUM.Y.
				

